# ERRATUM

**DOI:** 10.1590/0034-7167.20247701e03

**Published:** 2024-02-26

In the article “Nurses in the labor market: professional insertion, competencies and skills”, with DOI number: https://doi.org/10.1590/0034-7167-2016-0061, published in Revista Brasileira de Enfermagem, 2017;70(6):1220-6, on page 1225:

Include before REFERENCES:

**FUNDING**

This study was financed in part by the Coordenação de Aperfeiçoamento de Pessoal de Nível Superior - Brasil (CAPES) - Finance Code 001.

